# Prevalence and factors associated with malnutrition among school adolescents of Durame Town, Kambeta Tembaro Zone, Ethiopia

**DOI:** 10.11604/pamj.2023.44.163.27841

**Published:** 2023-04-06

**Authors:** Tariku Laelago Ersado, Tamiru Beyene Uliso, Teketel Ermias Geltore

**Affiliations:** 1 Department of Nursing, Wachemo University Durame Campus, Durame, Ethiopia,; 2 Department of Midwifery, Wachemo University Durame Campus, Durame, Ethiopia

**Keywords:** Malnutrition, thinness, stunting, overweight, underweight

## Abstract

**Introduction:**

malnutrition is a major public health issue affecting adolescents globally and developing countries in particular. Adolescent period is very sensitive to malnutrition. Adolescent malnutrition can be affected by different factors. The objective of this study was to assess prevalence and associated factors of adolescent malnutrition among high school students.

**Methods:**

an institutional-based cross-sectional study was carried out among school adolescents in Durame town high schools. Of the 498 selected school adolescents, 455 (91.4 %) were included in this study. Data were entered into Epi data and exported to World Health Organization (WHO) Anthroplus and Statistical package for the social sciences (SPSS). Odds ratio at 95% confidence interval (CI) was calculated to check for the existence and strength of association between variables. P-value less than 0.05 was used to declare significance of association.

**Results:**

the prevalence of stunting, thinness and overweight among adolescents was 26 (5.7%), 11(2.4 %) and 42 (9.2%), respectively. Residing in rural (AOR=5.31, 95% CI: 1.03-27.27) and utilize community based nutrition (AOR=0.26, 95% CI: 0.07-0.98) were associated with thinness. Male (AOR=3.55, 95% CI: 1.62-7.81) and having cattle (AOR=2.78, 95% CI: 1.393, 5.565) were associated with overweight.

**Conclusion:**

around two in twenty-five adolescents are stunted. About one in twenty-five adolescents are thin. More than two in twenty-five adolescents are overweight. Sex of adolescents, having cattle were associated with overweight of adolescent. Rural residence and utilizing community based nutrition were associated with thinness. Minister of Health and other health offices should implement nutrition education to school adolescents by giving emphasis on place of residence and for both male and female adolescents.

## Introduction

Adolescence is the phase of life between childhood and adulthood, from ages 10 to 19. It is a unique stage of human development and an important time for laying the foundations of good health. Adolescents experience rapid physical, cognitive and psychosocial growth. This period is very sensitive to malnutrition due to the increased physiologic need for nutrition. Adolescent malnutrition affects not only themselves but also their future generation [1]. Malnutrition, in all its forms, includes undernutrition (wasting, stunting, and underweight), inadequate vitamins or minerals, overweight, obesity, and resulting diet-related non-communicable diseases. The term malnutrition addresses 3 broad groups of conditions: undernutrition, which includes wasting (low weight-for-height), stunting (low height-for-age) and underweight (low weight-for-age); micronutrient-related malnutrition, which includes micronutrient deficiencies (a lack of important vitamins and minerals) or micronutrient excess; and overweight, obesity and diet-related non-communicable diseases (such as heart disease, stroke, diabetes and some cancers) [2]. Different studies conducted in different regions and countries showed variations in magnitude of malnutrition among adolescents. The study conducted in low- and middle-income counties showed that the prevalence of stunting (low height-for-age) and/or thinness (low Body mass index-for-age) among adolescents 15%. About 25% of adolescents were overweight or obese. A much smaller proportion of adolescents (2%) had concurrent stunting and overweight or obesity [3]. The study conducted in Allahabad showed the prevalence of underweight, overweight and obesity 3.9%, 12.1% and 5.4%, respectively [4]. The study done in Nigeria displayed the prevalence of underweight and overweight 20.1% and 3.2%, respectively [5]. The done in Abuja, Nigeria displayed the magnitude of wasting, stunting, overweight and obesity as 1.7%, 11.3%, 13.2%, and 2.6%, respectively [6]. The magnitude of adolescent malnutrition varied across Ethiopian studies. The study conducted in Sodo among school adolescents showed the overall prevalence of thinness (4.7%), stunting (5.2%), and overweight/obesity (5.0%) [7]. The study done in Northeast Ethiopia displayed the prevalence of stunting (26.5%) and thinness (58.3%) [8]. The Study conducted in Jimma displayed the prevalence of thinness, stunting, and overweight/obesity 11.6%, 15.6%, and 7.1%, respectively [9]. Other studies done in Dangila, Ethiopia identified the magnitude of stunting (24.8%) and thinness (7.1%) [10].

Adolescent malnutrition can be affected by different factors. Being male [4,7], parent education [11], income of household (HH) [9], availability of latrine [10] and number of family member greater than five [10] were some of the factors that affect adolescent malnutrition. Place of residence [11], age group, had nutrition and health information, living in food secured HH [12], being born from uneducated parents, occupation of parents, adolescents with low dietary diversity and adolescents attending government schools [13] were other factors that affected adolescent malnutrition. Malnutrition among adolescent can cause different bad consequences. Stunting begins in utero and manifests itself across infancy and affects brain and muscle growth. Stunting in adolescents is associated with impaired cognitive development and school achievement, and reduced economic productivity [14]. It is also associated with poor reproductive health outcomes in females [15]. Childhood stunting has also been reported to coexist with overweight or obesity at the individual level [16]. The consequences of concurrent stunting and obesity in adolescents are likely to compound health issues in adolescence and later in adulthood, particularly for females, given the heightened obstetric risk. Thinness in adolescence is associated with delayed maturation and poor muscle strength leading to constraints in capacity for physical work and reduced bone density later in life [17]. Obesity in adolescence has been associated with an increased risk of early onset of adult chronic diseases (type 2 diabetes, hypertension) and mortality in adult life [18]. Malnutrition, in all its forms, carries huge direct and indirect costs to individuals, families and to entire nations. The direct costs, such as the treatment of overweight- or obesity-related conditions, of undernutrition, including stunting, wasting and micronutrient deficiencies, have been estimated at between US$1 and US$2 trillion globally [19]. In addition, the direct costs of overweight and obesity-related non-communicable diseases were put at $1.4 trillion [19]. Additional costs are borne by families, in the form of higher medical bills, lost income due to illness, reduced school performance and later earnings due to cognitive impairment, funeral bills, and so on [20]. Since adolescent comprises about one fifth of worlds´ population number, they share high cost of malnutrition stated above. Most studies done in Ethiopia on adolescent malnutrition focused on only girls adolescents. However, this study included both boys and girls. The extent of adolescent malnutrition (undernutrition and over nutrition) is not well documented. Assessing prevalence and factors associated with adolescent malnutrition is important to avail updated information. This will be used as a baseline data for further research. In addition, it will help to design appropriate mechanism in preventing adolescent malnutrition. As to the researchers´ knowledge, there was no studies conducted so far on study area on adolescent malnutrition. Hence, this study identified the prevalence, and associated factors of adolescent malnutrition in Durame town administrative schools.

## Methods

**Description of study area:** the study was conducted in secondary schools of Durame town. The town is 125 km west of the regional capital city, Hawassa and 350 km south of Addis Ababa via Shashemene and 298 km via Hosanna. The total population of the town is 99,886 in 2020. Of the total population, male comprises 43,720 and female comprises 56,166. The economy of the town depends on agriculture (35%), civil worker (20%) and trade (45%). The means of transportation in the town is motorcycle, car, horse and foot transport administrations. The study was conducted from October 15 to 25, 2020. In Durame town, there are 51 different schools and 21 health facilities.

**Population:** the source of population of the study was school adolescents in Durame town in Kembata Tembaro zone. The study population was selected adolescents from selected high schools in Durame town Kembata Tembaro zone.

**Study design:** cross-sectional study design was employed.

**Sampling:** the sample size was calculated by using the formula for single population proportion for cross-sectional study and taking the proportion of thinness 11.6%, stunting 15.6% and overweight 7.1% [9] with confidence level of 95% and degree of precision of 4%. The total sample size was calculated with assumption of: 95% confidence interval (Z=1.96), d = 4% margin of error P = 0.156(P stands prevalence of stunting). The formula is


$$\frac{P\left(1-p\right)Z^{2}}{d^{2}}$$

The sample size was calculated for three malnutrition types (thinness, stunting and overweight) [21]. The sample size for stunting became the highest of others, which is 316. We considered a design effect of 1.5, this made the sample 474. We also added 5% for non-response. Hence, the final sample size became 498. Multistage sampling technique was used to select study participants.

**Data collection procedures:** structured questionnaire was employed to collect data. Face to face interviewer-administered questionnaire was used to collect data from each study subject. Nutritional status of adolescents was assessed by anthropometric measurements. The Questionnaire was first prepared in English and then, it was translated in to Amharic, and then it was translated back to English to check for its consistency. Four diploma nurses and two public health workers were recruited as interviewers and supervisors, respectively. Data collectors were trained for two days on questions included in the questionnaire, on interviewing techniques, purpose of the study, and importance of privacy, discipline and approach to the interviewees and confidentiality of the respondents. Data collection comprised socio-economic and demographic factors, nutrition and environmental factors, and Individual dietary diversity measurement. It also includes weight and height measurements of adolescents.

**Data quality control issues:** pretest was carried out on 5% of the total sample. Based on the result, data collectors were reoriented, and the questionnaire was modified. The principal investigators and supervisors made a day to day on site supervision during the whole period of data collection. At the end of each day, the questionnaires were reviewed and checked for completeness, accuracy and consistency by the supervisors and investigator and corrective discussion was taken with all the research team members. Remarks were given during morning times on how to eliminate or minimize errors and take corrective actions timely.

**Study variables:** the dependent variable of this study was adolescent malnutrition (stunting, thinness and overweight). The independent variables of this study included age of adolescents, place of residence, sex of adolescents, occupational status of father and mother, and educational status of father and mother. Other independent variables included HH income level, height, weight, eating habit, availability of sanitation service, meal skipping, dietary diversity, community based nutrient service, handwashing practice, and having latrine.

**Data analysis:** data were entered using Epi-Data version 3.1 and exported to SPSS version 20 and WHO Anthro software for analysis. World Health Organization Anthro software was used to assess nutritional status of adolescent. Height was measured in the standing position to the nearest 0.1 cm using a stable stadiometer with movable headpiece. Weight was measured using weighing scales to the nearest 10 g with light clothing. Anthropometric measurements were converted to height for-age Z score (HAZ) Body mass index (BMI)-for-age Z score (BAZ) using WHO Anthroplus software. Thinness is defined as BMI for-age Z-score less than -2 standard deviation from new WHO 2007 reference population [22]. Stunting is defined as height-for-age Z score less than -2 standard deviation of the new WHO 2007 reference population [22]. Overweight is defined as BMI-for-age Z-score between +1 and +2 standard deviation for new WHO 2007 reference population [22]. Individual dietary diversity measurement was done by asking twelve questions. Adolescents were asked whether they eat cereals, white roots/tubers, vegetables, fruit, meat, eggs, fish/seafood, legumes/nuts/seeds, dairy, oil/fats, sweet and spice in the last 24 hours. Those who consume 6 or more different types were categorized as having high dietary diversity and below 6 were categorized as having low dietary diversity [23]. Descriptive statistics (Frequency, mean, standard deviation, and proportion) were calculated to summarize the findings. Results were presented by tables and graphs. Binary logistic regression was used to predict variables which have independent association with outcome variables. Variables which have a significant association at p-value <0.25 in the bivariate analysis were taken to multivariate analysis to include all potential variables [24]. Odds ratio at 95% CI was calculated to check for the existence and strength of association between independent and dependent variable. P-value less than 0.05 was considered as statistical significant in the multivariate analysis.

**Ethics considerations:** ethical clearance was obtained from ethics review committee of Wachemo University Durame campus. Permission letter was obtained from Durame town educational office and school directors. During data collection, each respondent´s and teacher was informed about the purpose, scope and expected outcome of the research and appropriate informed oral consent was taken from the respondents. Written consent was obtained from teachers. In order to establish anonymous linkage, only the codes, not the names of the respondents, were registered on the questionnaire. During the training of data collectors and supervisors, ethical issue was addressed as an important component of the research. As the study was conducted in the midst of COVID-19, COVID prevention protocol like wearing mask, using hand sanitizer was strictly followed.

**Funding:** Wachemo University Durame campus funded the research. According to the research proposal, the funder ensured fulfilment of all the requirements from the design to dissemination of its results through institutional review board. The review board has made check-ups and reviews periodically as per the schedule. The University had no role in the study design, data collection and analysis, or preparation of the manuscript.

## Results

**Socio demographic and economic factors:** of the 498 selected school adolescent, 455 (91.4%) were participated in this study. The minimum and maximum age of the adolescents was 14 and 19 years, respectively. The mean age of adolescent was 16.92 years with standard deviation (SD) of 1.28. More than half, 234 (51.4%) adolescents were females. The place of residence showed that, 280 (61.5 %) adolescents were from urban areas. The occupation of fathers displayed that farmers were dominant group, 157 (34.5%) than others category. From occupation category of mothers, housewife accounted 283 (62.9%). Only, 40 (8.8%) fathers had no education while 76 (16.7%) of mothers had no education. The mean HH members were 6.6 with SD of 2.13. The minimum and maximum HH members were 2 and 17, respectively. For about 326 (71.8%) HH had 5 and more family members. More than half, 292 (64.2%) HH had cattle. Most adolescents (86.2%) were found in the age range of 16-19 years. Most of the adolescents (69.5%) consumed a meal three times in a day. The monthly income of HH was <100ETB for 17 (3.8 %) respondents while it was >4000 ET B for 355 (79.2%) respondents (Table 1).

**Table 1:** sociodemographic and economic character of the respondent, Durame, 2020

Variables	Frequency	%
**Sex (N=455)**		
Male	221	48.6
Female	234	51.4
**Place of residence(n=455)**		
Rural	175	38.5
Urban	280	61.5
**Occupation of father (n=454)**		
Government employee	152	33.4
Merchant	125	27.5
Farmer	157	34.6
Others*	20	4.4
**Occupation of mothers (n=450)**		
Housewife	283	62.9
Governmental employee	74	16.4
Merchant	92	20.4
Others**	1	0.2
**Education of fathers (n=454)**		
No education	40	8.8
Primary	141	31.1
Secondary	175	38.5
Tertiary	98	21.6
**Education of mothers (n=454)**		
No education	76	16.7
Primary	203	44.7
Secondary	129	28.4
Tertiary	46	10.1
**Have cattle**		
Yes	292	64.2
No	163	35.8
**Age of adolescents**		
14-15 years	63	13.8
16-19 years	392	86.2
**HH members (n=454)**		
less than 5	128	28.2
5 and above	326	71.8
**Monthly income (n=448)**		
< 1000	17	3.8
1000 to 2000	13	2.9
2001 to 3000	29	6.5
3001 to 4000	34	7.6
> 4000	355	79.2

* preacher, contractor, ** NGO

**Nutrition and environmental factors:** only 14 (3.1%) of HH had no latrine. About 410 (90.1%) adolescents washed their hands with soap before eating. Three hundred and thirteen (68.8 %) adolescents skipped the meal. Pipe was the most common source of drinking water, which accounted 353 (77.8 %). About 26.4% of adolescents were sick in the past 2 weeks prior to the survey. Only 106 (23.3 %) HH utilized community based nutrition. More than half (63.7%) adolescents had nutrition and health information. The mean number of feeding per day was 2.96 with SD of 0.59. The minimum and maximum number of feeding per day were 1 and 5, respectively (Table 2).

**Table 2:** nutritional and environmental factors, Durame, 2020

Variables	Frequency	%
**Availability of functional latrine at home (n=455)**		
Yes	441	96.9
No	14	3.1
**Wash hands with soap before eating (n=455)**		
Yes	410	90.1
No	45	9.9
**Skip meal (n=455)**		
Yes	313	68.8
No	142	31.2
**Source of drinking water (n=454)**		
Pipe	353	77.8
Spring	94	20.7
Other*	7	1.5
**Illness in the past 2 weeks prior to the survey (n=455)**		
Yes	120	26.4
No	335	73.6
**Utilize community based nutrition (n=455)**		
Yes	106	23.3
No	349	76.7
**Had nutrition and health information (n=455)**		
Yes	290	63.7
No	165	36.3
**Meal frequency per day (n=455)**		
1 to 2 times	76	16.7
3 times	316	69.5
4 times	63	13.8
**Individual dietary diversity**		
High dietary diversity	219	48.1
Low dietary diversity	236	51.9

* River, ground water

**Individual dietary diversity:** the result of this study displayed that 219 (48.1 %) and 236 (51.9 %) adolescents had high dietary diversity and low dietary diversity, respectively (Table 2).

### Prevalence of malnutrition among adolescent

**Anthropometric data:** the mean weight of adolescents was 56.6 with SD of 6.93 and ranging from 35 to 80 kg. The mean height of adolescents was 165.18 with SD of 8.99 and ranging from 120 to 186 cm. The prevalence of stunting among adolescent was 26 (5.7%) with 95% CI (3.7-7.9). The prevalence of thinness was 11 (2.4%) with 95% CI (1.1 -4) while the prevalence of overweight was 42 (9.2%) with 95% CI (6.8 -12.1).

### Factors associated with adolescent malnutrition

**Factors associated with stunting:** sex, occupation of father, education of father and mother were associated with stunting at bivariate association but no variables were associated with stunting at multivariate analysis.

**Factors associate with overweight:** sex of adolescents´, occupation of father, having cattle, meal frequency, skipping meal and individual dietary diversity were associated with overweight at bivariate analysis. However, in multivariate analysis only sex of adolescents and having cattle were associated with overweight. Male adolescents were 3 times more likely to be overweight than females (AOR=3.55, 95% CI: 1.62- 7.81). Adolescents who were from families who had cattle were 2.7 times more likely to be overweight (AOR=2.78, 95% CI: 1.39-5.56) (Table 3).

**Table 3:** factors associated with overweight, Durame, 2020

	Overweight			
Variables	Yes (N/%)	No (no/%)	COR (95 % CI)	AOR (95 % CI)
Sex of respondents				
Male	9(2)	212 (46.6)	3.867 (1.805,8.285)	3.55 (1.617,7.815)
Female	33 (7.2)	202( 44.2)		
**Occupation of father**		138	1	1
Government employee	14 (3.1)	138(30.4)	2.464 (0.723,8.396)	3.434 (0.920,12.811)
Merchant	10(2.2)	115 (25.3)	2.875 (0.806,10.258)	3.454 (0.897,13.297)
Farmer	14 (3.1)	143(31.5)	2.554 (0.750,8.697)	2.543 (0.676,9.563)
Others	4(0.9)	16(30.5)	1	1
**Have cattle**				
Yes	19 (4.2)	273 (60.0)	2.36 (1.24,4.48)	2.78 (1.393,5.565)
No	23 (5.0)	140 (30.8)	1	1
**Meal frequency per day**				
1 to 2 times	4 (0.9)	72 (15.8)	3.00 (0.877,10.259)	2.12 (0.572, 7.871)
3 times	29 (6.4)	287 (63.1)	1.65 (0.739,3.680)	1.41 (0.596, 3.345)
4 times	9 (2.0)	54 (11.9)	1	1
**Skip meal**				
Yes	35(7.7)	27 (61.1)	0.41 (0.178,0.951)	0.45 (0.191, 1.089)
No	(1.5)	13(29.7)	1	1
**Individual dietary diversity**				
High dietary diversity	2 (5.2)	19 (42.9)	0.67 (0.353,1.273)	0.62 (0.313, 1.233)
Low dietary diversity	1(4.0)	21 (47.9)	1	1

**Factors associated with thinness:** sex of adolescents, place of residence, education of father, utilize community based nutrition, having nutrition and health information and age of adolescents were associated with thinness at bivariate analysis. However, only place of residence and utilize of community nutrition were associated with thinness at multivariate analysis. Adolescents residing in rural areas were 5 times more likely to have thinness than urban adolescents (AOR=5.31, 95% CI: 1.03-27.27). Adolescents who were from HH who utilize community based nutrition were 74% less likely to have thinness (AOR=0.26, 95% CI: 0.07-0.97) (Table 4).

**Table 4:** factors associated with thinness, Durame, 2020

	Thinness			
Variables	Yes (N/%)	No (no/%)	COR (95 % CI)	AOR (95 % CI)
**Place of residence(n=455)**				
Rural	2 (0.4)	173 (38.0)	0.348 (0.074,1.630)	5.311 (1.034,27.272)
Urban	9 (2.0)	271 (59.6)	1	1
**Sex of respondent**				
Male	8 (1.8)	213 (46.8)	3469 (0.091,1.320)	0.309 (0.074,1.288)
Female	3 (0.7)	231 (50.8)	1	1
**Education of father(n=454)**				
No education	1 (0.2)	39 (8.6)	0.402 (0.025,6.589)	0.393 (0.021,7.358)
Primary	7 (1.5)	134 (29.5)	197 (0.024, 1.630)	0.229 (0.025, 2.060)
Secondary	2 (0.4)	173 (38.1)	0.892 (0.080,9.962)	1.047 (0.089,12.367)
Tertiary	1 (0.2)	97 (24.1)		
**Utilize community based nutrition**				
Yes	6 (1.3)	100 (22.0)	0.242 (0.072,0.810)	0.257 (0.068, 0.974)
No	5 (1.1)	344 (75.6)	1	1
**Had nutrition and health information**				
Yes	10 (2.2)	280 (61.5)	0.171 (0.022,1.346)	0.182 (0.022, 1.521)
No	1 (0.2)	164 (36.0)	1	1
**Age of adolescents**				
14-15 years	3 (0.6)	60 (13.2)	0.417 (0.108,1.615)	0.429 (0.092, 1.993)
16-19 years	8 (1.8)	384 (84.4)	1	1

## Discussion

This study tried to assess prevalence and associated factors of malnutrition among adolescents. The prevalence of stunting among adolescent was 26 (5.7%) with 95% CI (3.7-7.9). This is comparable with study done in Wolaita Sodo Town, which displayed the prevalence of stunting 5.2% [7]. But it is lower than study done in Lay Guyint (16.3%), Dangila (24.8%), Jimma (15.6%) and Ethiopia (20.7%) [8-10, 25]. The reason for the difference could be difference in geographic areas. The type of the foods grown in different areas can be varied, as a result the prevalence of the stunting can be varied. The prevalence of thinness was 11 (2.4%) with 95% CI (1.1 -4). This is lower than study of Wolaita Sodo (4.8%) and Lay Guyint (29%), Dangila (7.1%) and Jimma (11.6%) [7-10]. Other studies done in Jimma (29.2%), Ethiopia (27.5%) and Debra Tabor (4.9%) also displayed higher prevalence of thinness [25-27]. However, study done in Nigeria displayed lower prevalence of thinness (1.7 %) [6]. The difference could be difference in socioeconomic status, and geographical areas with dietary habit difference. Thinness in adolescence is associated with delayed maturation and poor muscle strength leading to constraints in capacity for physical work and reduced bone density later in life [17]. The prevalence of overweight was 42 (9.2%) with 95% CI (6.8 -12.1). This is higher than Wolaita Sodo (4.8%), Adama (3.3%) and Jimma (7.1%) studies [7,9,13]. It is also higher than the study of Bahidar, which displayed 8.4% [28]. But, it is lower than study of Nigeria, which showed the prevalence of overweight 20.1% [5]. The difference could be attributed by difference in socioeconomic and geographical variations. Habit of food intake, socioeconomic status and cultural variation between the study subjects could be a reason for variations of over-weight. Overweight in adolescence has been associated with an increased risk of early onset of adult chronic diseases (type 2 diabetes, hypertension) and mortality in adult life [18]. Male adolescents were more likely to be overweight than females. This is comparable with Allahabad [4] and Wolaita Sodo studies [7].

The reason for this could be girls may frequently report higher levels of weight-related concerns compared with boys, including desire to lose weight, feelings of guilt over eating too much and lower self-esteem [29,30]. In addition, the differences are likely a result of gender-based stereotypes as a feminine identity is typically characterized by eating smaller portions and preferring healthier options to maintain appearance, while a masculine eating identity is characterized by feeling full, with a focus on optimizing physical performance [31]. Adolescents who were from families who had cattle were more likely to be overweight. The reason for this could be having cattle are important source of milk. Adolescents from HH who had cattle may consume more milk and milk products. This in turn, can cause overweight. However, the current finding is contradicted with study from Sodo which showed that adolescent from no cattle HH were more likely to develop overweight [7]. Adolescents whose HH utilized community based nutrition were less likely to have thinness. This similar with study done in Amhara region [12]. The reason for this can be community based nutrition program can help to develop and strengthen the communities' capacity to assess and analyze the causes of their malnutrition problems and to take action by making better use of community and external resources to improve the nutritional status of HH. Adolescents residing in rural areas were more likely to have thinness than urban adolescents. This similar with study done in Lay Guyint woreda, Ethiopia [8]. It is also in line with study done Debra Tabor [27] and Mizan [32]. The reason for this could be urban residents have better nutrition and health information, access to safe water and sanitation facilities. This study has the following limitations. Factors that could affect adolescent malnutrition such as alcohol consumption and khat chewing were not included in this study. Questions that required a good memory were vulnerable to recall bias. As the study is cross-sectional, it neither characterizes seasonal difference of nutritional outcomes nor creates causal association. Hence, interpretation of the findings should consider the aforementioned factors.

## Conclusion

Malnutrition among adolescent is a common problem. Almost two in twenty-five adolescents are stunted. About one in twenty-five adolescents are thin. More than two in twenty-five adolescents are over weighed. Over weight among adolescents is most prevalent than stunting and thinness. This study identified the extent of under and over malnutrition in adolescents. As the extent of adolescent malnutrition is not well documented, this study provides data on dual adolescent malnutrition. This data will be used as baseline for further researches and policymakers can use this data to design strategies that focus on adolescent malnutrition. Government and non-government bodies working on human nutrition should work hard to end thinness among adolescents. Male adolescents were more overweight than female adolescents. The sex difference in adolescent malnutrition implies that adolescent nutrition intervention has to give equal emphasis for both male and females. These findings will help policymakers and programmers to design nutritional intervention that focused on both sexes. Having cattle in HH was determinant factors of overweight in adolescents. This finding implies that there may be inappropriate utilization of milk and milk products. Health workers can use this finding to educate communities on appropriate utilization of milk and milk products. Rural residents´ adolescents were thinner than urban residents´. Policymakers, health professional and other bodies working in adolescents malnutrition can use this finding to give due emphasis for rural residents adolescents. Adolescents whose HH utilized community based nutrition were less likely to have thinness. This finding implies that there is difference in nutrition access among HH who utilized community based nutrition and HH who do not utilized. Community health workers can use these findings to give emphasis for both HH who utilized and do not utilized community based nutrition. Minister of health and other health sectors offices need to implement nutrition education to school adolescents by giving emphasis on place of residence and for both male and female adolescents. Context-sensitive implementation and scale-up of interventions and policies for the double burden of malnutrition are needed to achieve the sustainable development goal to end malnutrition in all its forms by 2030.

### What is known about this topic

- *Adolescent malnutrition is a common problem in the world;*
- *There are different factors that can affect adolescent malnutrition*.

### What this study adds

- *Double burden of malnutrition was observed among school adolescents;*
- *Males adolescents were more likely to be overweight than females;*
- *Equal emphasis should be given for both male and female adolescents to combat malnutrition*.
